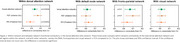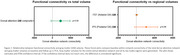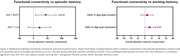# Posterior cortical atrophy: Reorganization of the dorsal attention network and its implications on volume loss and clinical performance

**DOI:** 10.1002/alz70856_099776

**Published:** 2025-12-24

**Authors:** Neha Singh‐Reilly, Jonathan Graff‐Radford, Mary M. Machulda, Christopher G Schwarz, David S. Knopman, Ronald Petersen, Clifford R. Jack, Val J Lowe, Keith A. Josephs, Jennifer L. Whitwell

**Affiliations:** ^1^ Mayo Clinic, Rochester, MN, USA; ^2^ Department of Neurology, Mayo Clinic, Rochester, MN, USA; ^3^ Department of Psychiatry and Psychology, Mayo Clinic, Rochester, MN, USA

## Abstract

**Background:**

In addition to visuospatial and visuoperceptual deficits, most PCA patients also report deficits in visual attention, episodic memory, and working memory. The dorsal attention network is known to play a critical role in modulating these clinical functions. However, little is known about the relationship of the dorsal attention network with other core networks and relationships to volume loss and memory functions in PCA.

**Method:**

Fifty‐seven PCA patients were recruited by the Neurodegenerative Research group, Mayo Clinic, and compared to 60 cognitively unimpaired (CU) individuals from the Mayo Clinic Study of Aging. The CONN functional connectivity toolbox was used to calculate within‐ and between‐network connectivity. Functional connectivity within the key regions (i.e., inter‐hemisphere connectivity) of the dorsal attention network, namely frontal eye field (FEF) and intraparietal sulcus (IPS), and the entire network was generated. Functional connectivity between the dorsal attention network and the frontoparietal, visual, and default mode networks (DMN) were also generated using CONN's network parcellation atlas. Multivariate linear regression models were fit i) to compare within‐ and between‐network connectivity between both groups and ii) to assess relationships between within‐network connectivity and both gray matter volumes and clinical test scores.

**Result:**

PCA showed reduced within‐network connectivity in the dorsal attention network, specifically within the IPS and the entire network compared to CU. An increase in between‐network connectivity between the frontoparietal network and both FEF and the entire dorsal attention network was noted in PCA compared to CU (Figure 1). Lower dorsal attention within‐network connectivity was associated with a trend for lower volumes in the entire network (Figure 2) and significantly lower scores on the auditory verbal learning test‐recognition percent correct (AVLT‐RCP) and Wechsler Memory Scale (WMS) III‐digit span backward in PCA (Figure 3).

**Conclusion:**

These findings suggest that within‐ and between‐network connectivity of the dorsal attention network, both within key regions and the entire network, was disrupted in PCA patients. However, this network did not show disrupted connectivity with the core PCA networks. Within‐network connectivity disruptions in the dorsal attention network are associated with aspects of memory function, and to some extent, with volume loss.